# Commentary: “*TRIB3* promoter 33 bp VNTR is associated with the risk of cerebrovascular disease in type 2 diabetic patients”

**DOI:** 10.3389/fgene.2026.1802422

**Published:** 2026-03-18

**Authors:** Benedetta Torbidoni-Baldassari, Lucrezia Romagnoli, Valerio Napolioni

**Affiliations:** 1 Genomic and Molecular Epidemiology (GAME) Lab, School of Biosciences and Veterinary Medicine, University of Camerino, Camerino, Italy; 2 International School of Advanced Studies, University of Camerino, Camerino, Italy

**Keywords:** genetics, polymorphism, TRIB3, variable number of tandem repeat, VNTR

We read with interest the article by Lai et al. (2022) on the association of the 33bp Variable Number of Tandem Repeat (VNTR) in the *TRIB3* promoter region with the risk of cerebrovascular disease. While the study presents intriguing findings, we believe several important concerns warrant further discussion.

For instance, as the authors state, the VNTR in the *TRIB3* promoter could affect TRIB3 transcription. As the authors also note, a previous study by Örd et al. (2020) found that individuals carrying the SNP allele associated with increased TRIB3 mRNA expression for 3 *TRIB3* eQTL SNPs (rs62191440, rs6076475, rs3818193) – largely independent of each other (LD R^2^ < 0.3) – also tend to have a higher *TRIB3*-VNTR repeat count. This finding suggests that the VNTR may underlie differences in TRIB3 expression levels.

To date, the work by Lai et al. (2022) is only the second-ever published article on *TRIB3*-VNTR, following the initial report by Örd et al. (2020). Our first concern is the lack of clarity in the variant classification used by Lai and colleagues (Lai et al., 2022). Indeed, Örd et al. (2020), classified the VNTR polymorphism in the global population by naming the alleles based on the number of repeats, suggesting five alleles: *1, *2, *3, *4, *5. This classification is only briefly mentioned in the work of Lai et al. (2022), even though it is heavily implied in the Supplementary materials. This lack of clarity is more evident when considering the paragraph “Statistical analysis” in the section “Materials and methods”. Here, it is reported that the authors “*divided patients into three groups based on the total number of TRIB3 33bp tandem repeats in both alleles: low number of 33bp tandem repeats (repeats ≤ 6), intermediate number of 33bp tandem repeats (6<repeats ≤ 8) and high number of 33bp tandem repeats (repeats > 8)*”. The sentence’s phrasing is ambiguous, leading readers to assume that the number of repeats in the individual’s genotype was summed to classify the individual into one of the three groups mentioned, even though it does not state this explicitly.

Our most serious concern arises from the classification method used by Lai and colleagues (Lai et al., 2022). As a matter of fact, classifying individuals by summing the number of tandem repeats that make up that individual’s VNTR genotype is unconventional at best. This type of classification could assign the same functional role to genotypes that may have different effects on phenotype expression, as, for example, would happen with the 2/5 and 3/4 genotypes. While we acknowledge this classification could be appealing for statistical power considerations, this approach assumes a linear dose-response effect, which has not been established for this specific locus, and may overlook non-additive dynamics, such as threshold effects or allele-specific regulatory mechanisms.

In addition to the concerns stated above, we compared the frequencies of the VNTR allele reported by Örd et al. (2020) with those reported by Lai et al. (2022), using the Han and Southern Han populations from Örd et al. (2020), as the Han population is the major ethnic group in China (Press Release, 2026). Table 1 presents comparisons between Örd et al. (2020) and Lai et al. (2022) for allele frequencies. As shown, the comparison revealed no significant difference in allele frequency after Fisher’s exact test. Furthermore, we checked the Hardy-Weinberg Equilibrium, which showed no discrepancy (P > 0.999).

**TABLE 1:** Allele frequency comparisons. The last column reports the p-value from Fisher’s exact test. The comparison was carried out performing the test Lai et al. (2022) vs. Örd et al. (2020) – Han population Lai et al. (2022), vs. Örd et al. (2020) - Northern Han population, and Lai et al. (2022) vs. Örd et al. (2020) - all Han populations.

Study	Alelle frequency (%)					Population allele count	Fisher’s exact test p-value
	*1	*2	*3	*4	*5		
Örd et al. (2020) - Han population	0 (0.0)	11 (18.3)	32 (53.3)	1 (1.7)	16 (26.7)	60	0.119
Örd et al. (2020) - Northern Han population	0 (0.0)	3 (16.7)	11 (61.1)	0 (0.0)	4 (22.2)	18	0.536
Örd et al. (2020) - all Han populations	0 (0.0)	14 (18.0)	43 (55.1)	1 (1.3)	20 (25.6)	78	0.066
Lai et al. (2022)	1 (0.1)	136 (8.5)	939 (58.8)	70 (4.4)	450 (28.2)	1,596	---

The analyses described were deemed necessary to convey important information in genetic association studies (Napolioni, 2014), especially when dealing with newly discovered and still poorly characterized VNTRs.

While the study by Lai et al. (2022) provides valuable preliminary evidence supporting a potential role of the *TRIB3* promoter VNTR in cerebrovascular risk among individuals with type 2 diabetes, the variant classification strategy adopted raises important methodological concerns. Providing clearer definitions of alleles, along with a classification system that accounts for both allele-specific and potentially non-additive effects, would enhance the interpretation of results and facilitate comparisons with the existing literature. Given the limited number of studies currently available on this locus, careful standardization of analytical approaches will be essential to advance our understanding of the functional and clinical relevance of the *TRIB3*-VNTR.
